# Plastome evolution and phylogenomics of Trichosporeae (Gesneriaceae) with its morphological characters appraisal

**DOI:** 10.3389/fpls.2023.1160535

**Published:** 2023-05-09

**Authors:** Yan-Fang Cui, Peng Zhou, Kun-Li Xiang, Qiang Zhang, Hua Yan, Li-Guo Zhang, Bo Pan, Yu-Song Huang, Zhi-You Guo, Zhen-Yu Li, Xiao-Guo Xiang

**Affiliations:** ^1^ Jiangxi Province Key Laboratory of Watershed Ecosystem Change and Biodiversity, Institute of Life Science and School of Life Sciences, Nanchang University, Nanchang, Jiangxi, China; ^2^ State Key Laboratory of Systematic and Evolutionary Botany, Institute of Botany, Chinese Academy of Sciences, Beijing, China; ^3^ Guangxi Key Laboratory of Plant Conservation and Restoration Ecology in Karst Terrain, Guangxi Institute of Botany, Guangxi Zhuang Autonomous Region and Chinese Academy of Sciences, Guilin, China; ^4^ Qiannan Normal College for Nationalities, College of Biological Sciences and Agriculture, Duyun, Guizhou, China

**Keywords:** Gesneriaceae, *Hemiboea*, molecular markers, phylogenomics, plastome evolution, Trichosporeae

## Abstract

Trichosporeae is the largest and most taxonomically difficult tribe of Gesneriaceae due to its diverse morphology. Previous studies have not clarified the phylogenetic relationships within this tribe on several DNA markers, including the generic relationships within subtribes. Recently, plastid phylogenomics have been successfully employed to resolve the phylogenetic relationships at different taxonomic levels. In this study, plastid phylogenomics were used to explore the relationships within Trichosporeae. Eleven plastomes of *Hemiboea* were newly reported. Comparative analyses, phylogeny and morphological character evolution within Trichosporeae were conducted on 79 species representing seven subtribes. The *Hemiboea* plastomes range from 152,742 bp to 153,695 bp in length. Within Trichosporeae, the sampled plastomes range from 152,196 bp to 156,614 bp and GC content from 37.2% to 37.8%. A total of 121–133 genes were annotated in each species, including 80–91 protein-coding genes, 34–37 tRNA genes, and 8 rRNA genes. The contraction and expansion of IR borders were not detected, and gene rearrangements and inversions did not occur. The 13 hypervariable regions were proposed as the potential molecular markers for species identification. A total of 24,299 SNPs and 3,378 indels were inferred, and most of the SNPs were functionally missense and silent variations. There were 1968 SSRs, 2055 tandem repeats and 2802 dispersed repeats. The RSCU and ENC values indicated that the codon usage pattern was conserved in Trichosporeae. Both the phylogenetic frameworks based on the whole plastome and 80 CDSs were basically concordant. The sister relationships between Loxocarpinae and Didymocarpinae were confirmed, and *Oreocharis* was a sister group of *Hemiboea* with high support. The morphological characters showed a complex evolutionary pattern of Trichosporeae. Our findings may contribute to future research on genetic diversity, morphological evolutionary patterns, and conservation of the tribe Trichosporeae.

## 1 Introduction

Gesneriaceae comprises *ca*. 150 genera and 3000 species and mainly distributes in the tropics and subtropics of the world (Li and Wang, 2005; Möller et al., 2016). These plants are usually perennial herbs with gorgeous flowers and various leaves, which have high ornamental value and great potential for garden and horticultural applications (Li and Wang, 2005). For example, *Sinningia speciosa* Benth. & Hook., *Episcia cupreata* (Hook.) Hanst. and *Streptocarpus ionanthus* (H. Wendl.) Christenh. have become famous ornamental flowers. Furthermore, many Gesneriaceae species, such as *Corallodiscus lanuginosus* (Wall. ex R. Br.) B.L. Burtt, *Dorcoceras hygrometricum* Bunge, *Hemiboea subcapitata* Clarke, *Lysionotus pauciflorus* Maxim. and *Primulina eburnea* (Hance) Yin Z. Wang, have been used as folk medicines in China for a long time (Li and Wang, 2005). As the largest tribe of Gesneriaceae, Trichosporeae includes 10 subtribes and distributes in tropical and subtropical Asia, Europe and Africa. Its morphological characters are diverse, and traditional classifications based on morphology have identified genera that belong to different alliances and geographical groups. This makes it the most taxonomically difficult tribe (e.g., Weber et al., 2013; Möller et al., 2016). Phylogenetically, the tribe is subdivided into distinct clades, but their relationships are not fully resolved and not consistently well supported (e.g., Wang et al., 2010; Möller et al., 2011; Li et al., 2022). Based on 16 plastid, 9 nuclear and 1 mitochondrial DNA makers, Roalson and Roberts (2016) showed that Streptocarpinae and Loxocarpinae were sister groups, while Li et al. (2022), based on 11 chloroplast regions and nuclear ITS, indicated that Loxocarpinae was a sister group to Didymocarpinae. Besides, the phylogenetic positions of some genera within Didymocarpinae were also uncertain. For example, both Roalson and Roberts (2016) and Li et al. (2022) indicated that *Hemiboea* was close to *Lysionotus* with weak supporting values, while Hsieh et al. (2022) supported that *Oreocharis* was a sister group of *Hemiboea* based on plastomes with one IR excluded. Möller et al. (2011) proposed to employ more molecular markers, especially conserved plastome sequences, to improve the phylogenetic relationships within Trichosporeae. Chen et al. (2020) also intensively suggested adopting more DNA sequences to resolve the phylogeny of Gesneriaceae. Therefore, developing more effective molecular markers is crucial for the conservation and utilization of Trichosporeae as well as Gesneriaceae.

With the advancement of high-throughput sequencing technologies, more and more plastomes are being successfully sequenced and annotated (Dobrogojski et al., 2020). Most angiosperms plastomes are circular with four regions: a large single copy (LSC) region, a small single copy (SSC) region and two copies of inverted repeats (IRa/b), with a length ranging from 120–160 kb with 30–40% GC content (Mower and Vickrey, 2018). The high conservatism and slow evolution rate of plastomes make them suitable for inferring phylogenetic relationships (e.g., Lu et al., 2016; Chi et al., 2018; Lee et al., 2019). Rosaceae is one of the taxonomically difficult lineages with hybridization and rapid radiation, and the phylogenetic relationships among subfamilies, tribes and genera have been successfully resolved by employing plastid phylogenomics (Zhang et al., 2017). Wei et al. (2021) clarified the phylogenetic relationships within *Euphorbia* based on plastomes, which have highly homogenous morphological characters, and revealed that it may have undergone complex plastome evolution. Overall, plastome data has a high power to explore the phylogenetic relationships of different taxonomic levels.

In this study, we newly sequenced, assembled, and annotated plastomes of 11 *Hemiboea* species, and combined them with 68 previously reported plastomes of Trichosporeae, we conducted comparative analyses, phylogenetic reconstruction and morphological characters appraisal within Trichosporeae. Our objectives are: (1) to investigate general plastome features and sequence divergence in Trichosporeae; (2) to identify the most variable regions as potential DNA barcodes for future species identification within Trichosporeae; (3) to explore the phylogenetic relationships within this tribe; (4) to assess the morphological evolution of Trichosporeae. Our study provides abundant information for the phylogeny, biogeography, and conservation of Trichosporeae.

## 2 Materials and methods

### 2.1 Sampling, sequencing, assembly, and annotation

In this study, eleven new plastomes of *Hemiboea* species were obtained. Sixty-eight published plastomes of Trichosporeae were downloaded from GenBank and updated the annotations (one species of *Actinostephanus*, two species of *Beccarinda*, one species of *Boeica*, one species of *Corallodiscus*, one species of *Dorcoceras*, one species of *Haberlea*, one species of *Henckelia*, one species of *Leptoboea*, one species of *Litostigma*, one species of *Lysionotus*, five species of *Oreocharis*, 12 species of *Paraboea*, four species of *Petrocodon*, 28 species of *Primulina*, two species of *Rhynchotechum*, one species and five varieties of *Streptocarpus*), representing seven subtribes (Corallodiscinae, Didymocarpinae, Leptobaeinae, Litostigminae, Loxocarpinae, Ramondinae and Streptocarpinae). Additionally, two species of Gesnerioideae (*Achimenes cettoana* H.E. Moore and *Achimenes erecta* (Lam.) H.P. Fuchs) were selected as outgroups. The detailed information of samples was listed in **Table S1**.

Total DNA was extracted from silica gel-dried leaves using the modified CTAB method (Doyle and Doyle, 1987). Library construction was performed with the NEB Next^®^ Ultra DNA Library Prep Kit (NEB, USA) following the manuals. Libraries for paired-end 150 bp sequencing were conducted using an Illumina HiSeq 2000 platform to generate approximately 4 Gb of data for each sample. The library preparation and sequencing were finished at the Kunming Institute of Botany, Chinese Academy of Sciences (Yunnan, China). The quality of raw sequence reads was assessed in FastQC v0.11.9 (Brown et al., 2017) and the adapters and low-quality reads were filtered in Trimmomatic v0.39 (Bolger et al., 2014). Then, the clean reads were assembled using GetOrganelle v1.7.3.2 with default parameters (Jin et al., 2020). The assembled genomes were checked and visualized in Bandage v0.7.1 (Wick et al., 2015). Finally, the plastomes were annotated and manually checked in Geneious v9.05 (Kearse et al., 2012) with *Hemiboea ovalifolia* (W.T. Wang) A. Weber & Mich. Möller (NC_054358) as a reference. The physical map was drawn using OGDRAW (https://chlorobox.mpimp-golm.mpg.de/OGDraw.html).

### 2.2 Structure and sequence divergence analysis

The expansion and contraction of IR boundary were visualized in IRscope v3.1 (Amiryousefi et al., 2018). Sequence alignments were performed using MAFFT v7 (Katoh and Standley, 2013) and manually adjusted in Geneious v9.05 (Kearse et al., 2012). Before the collinearity analysis, one copy of the IR region was removed. Possible rearrangements and inversions were detected using the Mauve v1.1.3 (Darling et al., 2010) plugin in Geneious v9.05 (Kearse et al., 2012). To further detect hypervariable regions within Trichosporeae, nucleotide diversity (pi) of protein-coding genes (CDSs) and non-coding regions were evaluated using DnaSP v6.12.03 (Rozas et al., 2017). Functional annotations for the nucleotide variations within Trichosporeae plastomes were performed using snpEff v5.1 (Cingolani, 2012).

### 2.3 Repetitive sequence analysis

Three types of repeats sequences, including simple sequence repeats (SSRs), tandem repeats, and dispersed repeats, were analyzed. SSRs were detected using MISA v2.1 (Beier et al., 2017) and visualized using R packages ggpubr and ggplot2 (Wickham, 2016; Kassambara, 2023). Tandem repeats were performed using Tandem Repeats Finder v0.9 (Benson, 1999). The identification of dispersed repeats (including forward, reverse, complement, and palindromic) was analyzed in REPuter (Kurtz et al., 2001) following the parameter settings in Cauz-Santos et al. (2020).

### 2.4 Codon usage analysis

To quantify the degree of the codon usage bias, both the relative synonymous codon usage (RSCU) ratio and the effective number codons (ENC) for 79 Trichosporeae plastomes were estimated using CodonW v1.4.2 (Peden, 1999). The RSCU>1 indicates a preferred codon, while the RSCU<1 indicates the opposite. The ENC values range from 20 (where only one synonymous codon is used to encode amino acids) to 61 (where every synonymous codon is used equally). The ENC<35 indicates that a gene has significant codon bias (Tao and Yao, 2020).

### 2.5 Phylogenetic analysis

The phylogenetic relationships were inferred from whole plastome sequences (excluding one IR) and concatenation of 80 CDSs. Before performing maximum likelihood (ML) and Bayesian inference (BI), we calculated the best nucleotide substitution model for each dataset using jModelTest2 (Darriba et al., 2012) under the Akaike information criterion. We conducted ML analyses in RAxML v8.2.12 (Stamatakis, 2014) with a rapid bootstrap analysis (1000 replicates) and searching for the best-scoring tree simultaneously. BI analyses were performed in MrBayes v3.2.6 (Ronquist et al., 2012) with four Markov Chain Monte Carlo chains starting with a random tree. We ran 2,000,000 generations and sampled every 1000 generations. The convergence was assessed in Tracer v1.7 (Rambaut et al., 2018). The majority rule (>50%) consensus tree was obtained after removing the “burn-in” samples (the first 20% of the sampled trees).

### 2.6 Ancestral state reconstruction of morphological characters

Following Li and Wang (2005); Wang et al. (1998), and Weber et al. (2013), nine morphological characters and their states were selected to study their evolution within Trichosporeae. These characters were habit (perennial herb, annual herb, shrub), stem (rhizome, creeping stem, erect stem), leaf margin (dentation, entire, undulate, cleft), inflorescence (cymes, solitary), number of stamens (2, 4), number of staminodes (0, 1, 2, 3), capsule (twisted, not twisted), capsule dehiscing type (valves 2, valves 4, berry), and seeds (appendaged, unappendaged). Ancestral state reconstruction were performed under the ER model (equal-rates model) using R packages phytools (Revell, 2012).

## 3 Result

### 3.1 Plastome features and gene contents

**Figure 1:**
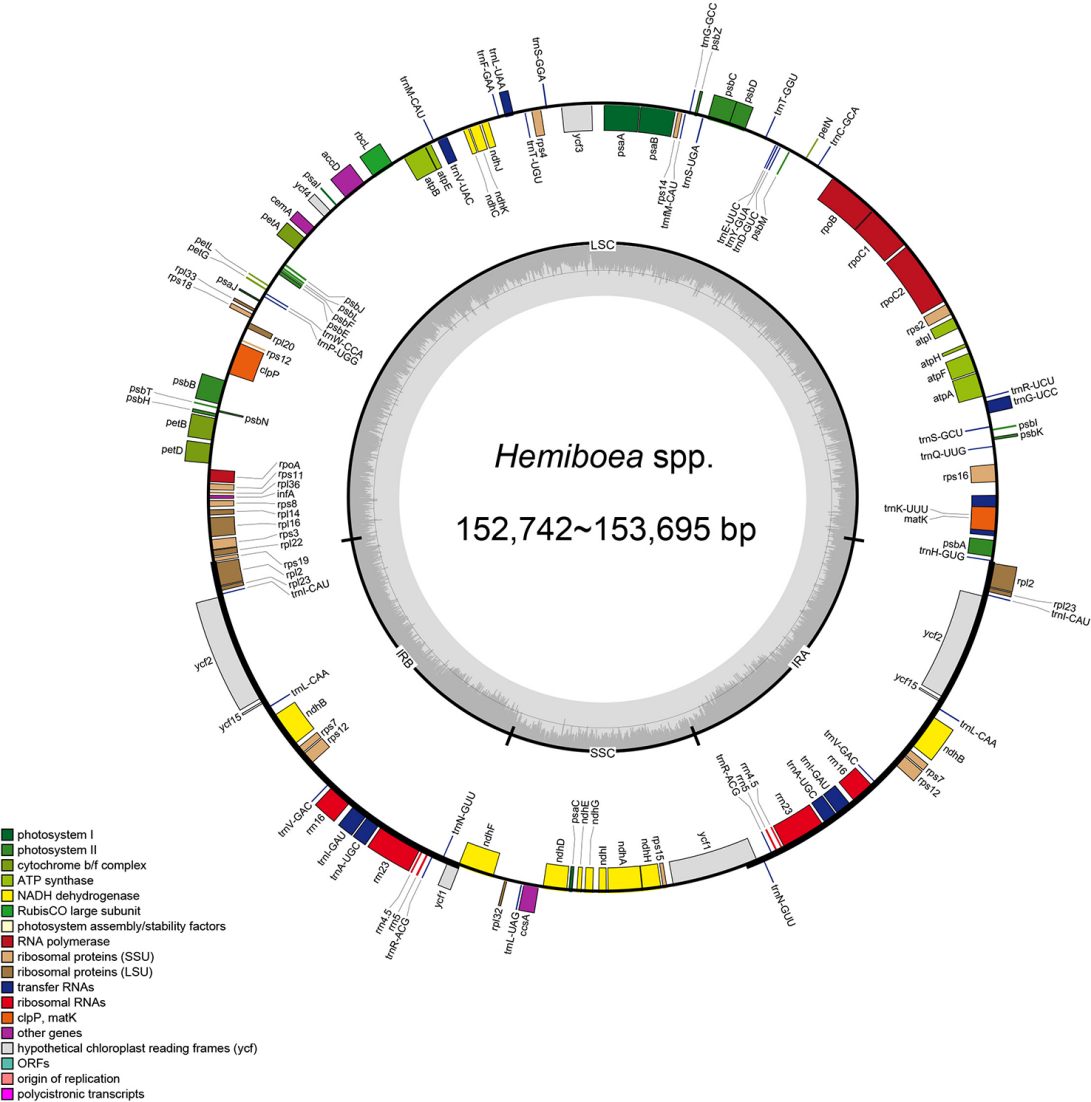
Plastome structure of 11 *Hemiboea* species. Bars of different colors indicate different functional groups. LSC, large single copy region; SSC, small single copy region; IRa and IRb, two inverted repeat regions.

The plastomes of the 11 *Hemiboea* species displayed the typical quadripartite structure (**Figure 1**). The genome sizes ranged from 152,742 bp (*H. purpurea* Yan Liu & W.B. Xu) to 153, 695 bp (*H. fangii* Chun) (**Table S2**). The plastomes contained a pair of IRs (25,432–25,494 bp), LSC region (83,724–84,670 bp), and SSC region (17,788–18,256 bp). The GC content of the plastomes ranged from 37.5% to 37.6%. The GC content of the IR region ranged from 43.1% to 43.2%, the GC content of the LSC region ranged from 35.5% to 35.6%, while the GC content of the SSC region ranged from 30.9% to 31.2% (**Table S2**). Each *Hemiboea* plastome consisted of 132 genes, comprising 87 CDSs, 37 transfer RNA (tRNA) genes, and eight ribosomal RNA (rRNA) genes.

The total length of the 79 plastomes in the tribe Trichosporeae ranged from 152,196 bp (*Primulina suichuanensis* X. L. Yu & J. J. Zhou) to 156,614 bp (*Corallodiscus lanuginosus* (Wallich ex R. Brown) B. L. Burtt) (**Table S2**). All the plastomes also displayed a typical quadripartite structure, including LSC region (83,063–87,429 bp), SSC region (17,478–18,330 bp) as well as a pair of IRs regions (25,272–25,657 bp) (**Table S2**). The total GC content ranged from 37.2% to 37.8%. The GC content of the IR region ranged from 43.1% to 43.2%. The GC content of the LSC region ranged from 34.9% to 35.8%, while the GC content of the SSC region ranged from 30.9% to 31.7%. The 79 Trichosporeae plastomes contained 121–133 genes, including 80–91 CDSs, 34–37 tRNAs, and eight rRNAs (**Table S2**). Among these genes, six CDSs (*ndhB, rpl2, rpl23, rps7, ycf15*, and *ycf2*), seven tRNA (*trnA*-*UGC*, *trnI*-*CAU*, *trnI*-*GAU*, *trnL*-*CAA*, *trnN*-*GUU*, *trnR*-*ACG*, and *trnV*-*GAC*), four rRNA (*rrn16*, *rrn23*, *rrn4.5* and *rrn5*) were duplicated in the IR regions. The gene *rps12* contained three exons, two of which were duplicated in the IRs. Further, 16 genes (*ndhA, ndhB, petB, petD, atpF, rpl16, rpl2, rps12, rps16, rpoC1*, *trnG*-*UCC*, *trnA*-*UGC*, *trnI*-*GAU*, *trnK*-*UUU*, *trnL*-*UAA*, and *trnV*-*UAC*) had one intron, and two genes (*clpP* and *ycf3*) had two introns (**Table S3**).

### 3.2 Plastome structural variations and sequence divergence

**Figure 2:**
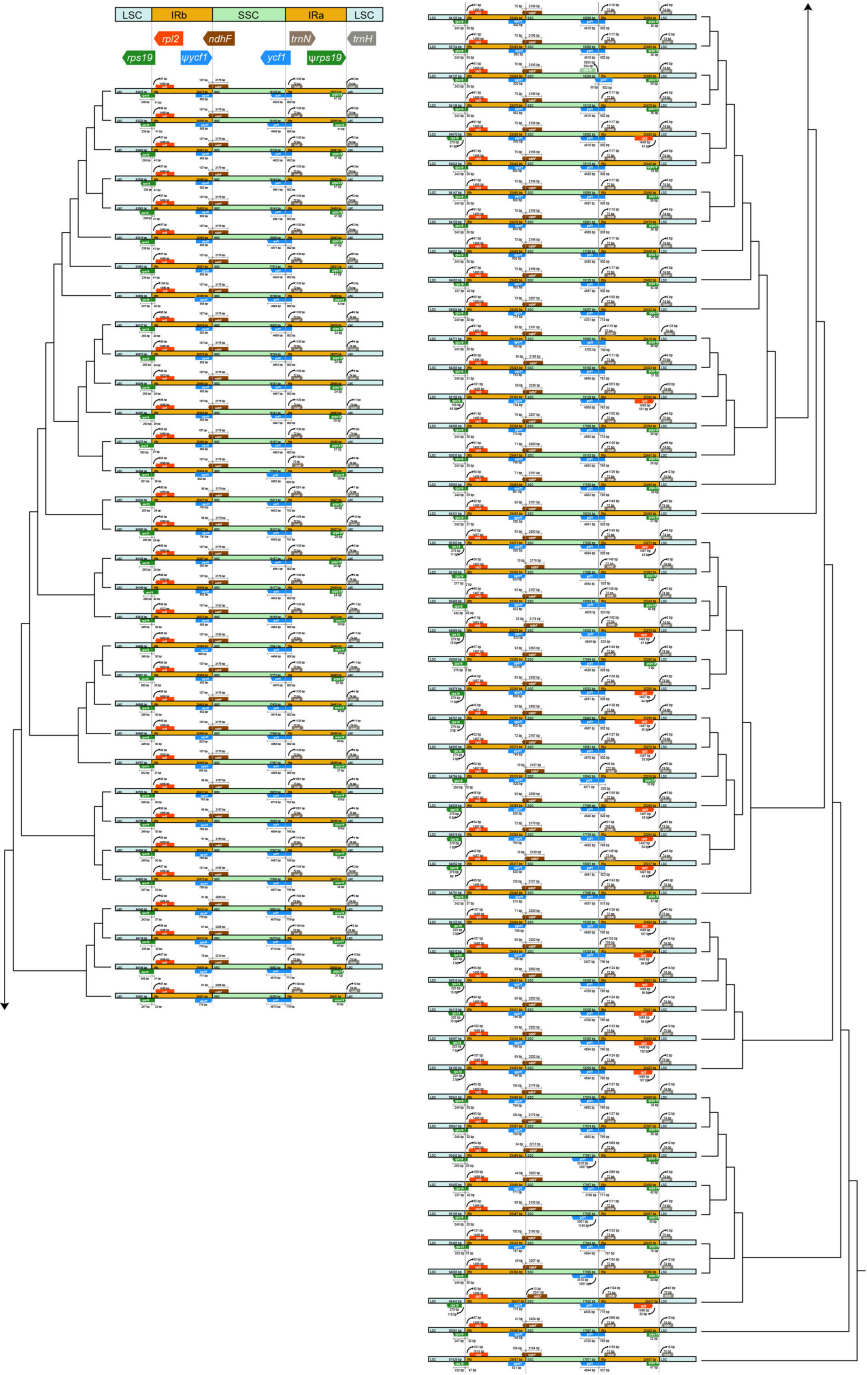
Visualization of the expansion and contraction in the inverted repeat region (IR) boundary. The topology was the ML tree based on plastomes with one IR excluded.

The expansion and contraction in the IR/SC boundary regions were shown in **Figure 2**. Within Trichosporeae, *rpl2* and *rps19* genes located in the LSC-IRb borders. Although the length of *rps19* was 179 bp, its location showed differences. In 62 species, *rps19* crossed the border, leading to the appearance of a ψ*rps19* in IRa-LSC borders. The *ndhF* located across the IRb-SSC borders, except for *Haberlea rhodopensis* Friv. The SSC-IRa borders located in *ycf1* gene. The *ycf1* gene crossed the border in 76 species, leading to the appearance of a ψ*ycf1* in IRa-LSC borders. However, the *ycf1* gene in *Boeica ferruginea* Drake, *Leptoboea multiflora* (Clarke) Clarke, and *Beccarinda cordifolia* (J. Anthony) B.L. Burtt was completely located in the SSC region. The IRa-LSC borders were between the ψ*rps19*, *rpl2*, and *trnH*.

**Figure 3:**
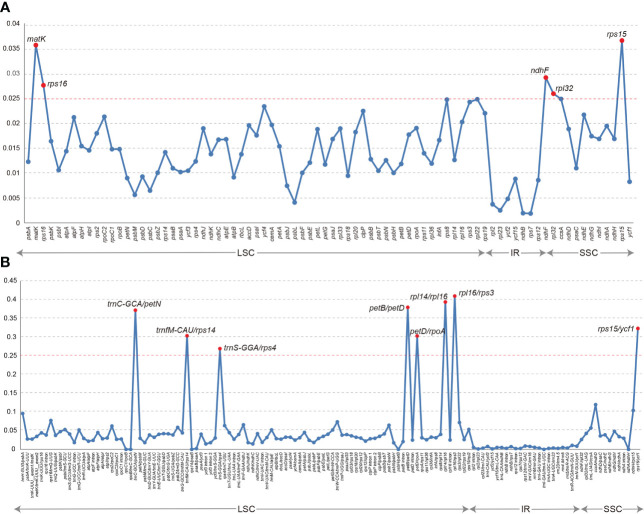
Comparison of nucleotide diversity (Pi) values in Trichosporeae plastomes. **(A)** Pi values of CDSs, **(B)** Pi values of non-coding regions.

The collinearity analysis revealed no gene rearrangements or inversions in the Trichosporeae plastomes (**Figure S1**). The pi of the CDSs ranged from 0.001876 (*rps7*) to 0.036559 (*rps15*). Notably, five CDSs (*rps15, matK, ndhF, rps16*, and *rpl32*) exhibited high pi values (pi>0.025; **Figure 3A**). Among the non-coding regions, the pi values ranged from 0 (i.e., *rpoC1/rpoB, trnI-GAU/trnA-UGC* and *ndhA/ndhH*) to 0.409580 (*rpl16/rps3*). Eight non-coding regions (i.e., *rpl16/rps3*, *rpl14/rpl16*, *petB/petD*, *trnC-GCA/petN*, *rps15/ycf1*, *trnfM-CAU/rps14*, *petD/rpoA* and *trnS-GGA/rps4*) showed significantly high pi values (pi>0.25; **Figure 3B**). The aligned matrix of the 79 Trichosporeae plastomes contained 24,299 single nucleotide polymorphisms (SNPs) and 3378 insertion-deletions (indels). The majority of SNPs from coding genes were functionally missense variations, while 6448 SNPs (43.20%) and 104 SNPs (0.70%) were silent (synonymous) and nonsense variations, respectively (**Table 1**).

**Table 1:** Functional annotaitons for the single-nucleotide variants (SNVs) detected in the Trichosporeae plastomes.

Region	Functional class	Variation type	Count
Exon	Missense		8,375
		Missense variant	8,309
		Stop lost	66
	Nonsense		104
		Stop gained	104
	Silent		6,448
		Stop_retained variant	98
		Synonymous variant	6,350
Intron		Intron variant	22,712
Intergenic		Intergenic region	12,439
downstream		Downstream gene variant	128,209
upstream		Upstream gene variant	122,523

**Figure 4:**
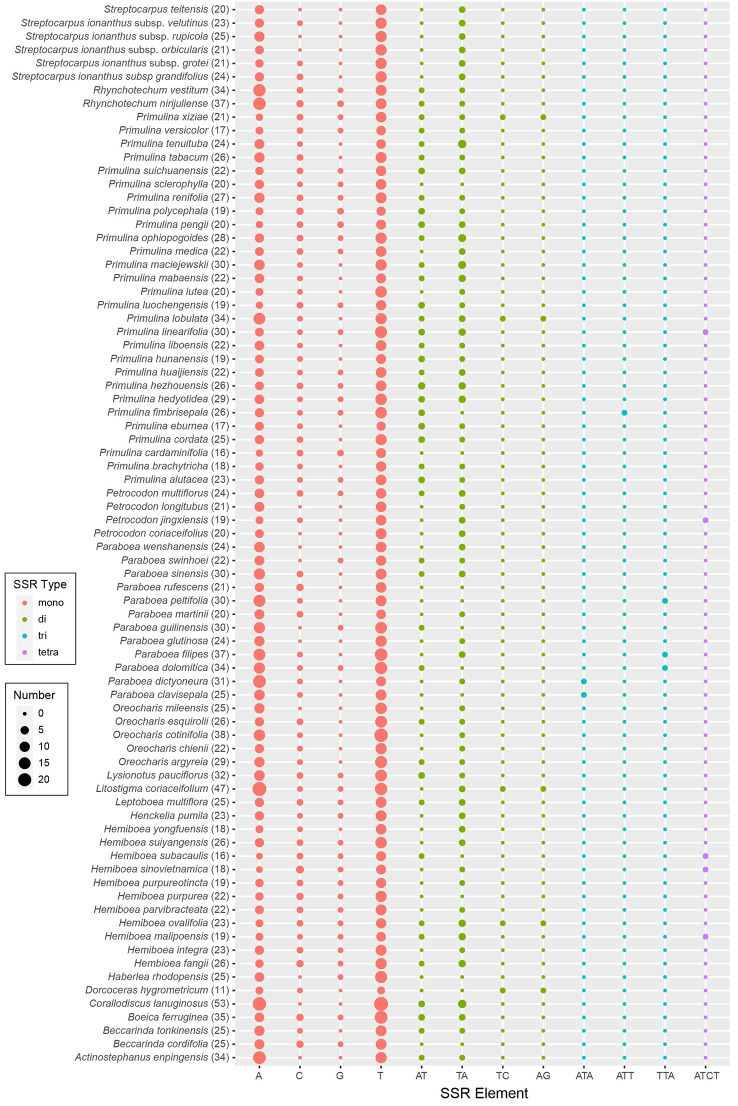
Plot of each SSR repeat pattern numbers of 79 Trichosporeae plastomes with one IR excluded. The total number of SSRs in each plastome is shown in parentheses.

The results of SSRs, tandem repeats, and dispersed repeats in the 79 Trichosporeae plastomes were shown in **Figure 4** and **Table S4**. The number of identified SSRs ranged from 11 (*Dorcoceras hygrometricum*) to 53 (*Corallodiscus lanuginosus*). A total of 1968 SSRs were detected in the 79 Trichosporeae plastomes, of which 1432 SSRs (62.60%) located in the LSC region, 280 SSRs (14.23%) in the SSC region, and 258 SSRs (13.11%) in the IR regions. Four types of SSRs (mono-nucleotide, di-nucleotide, tri-nucleotide and tetra-nucleotide) were identified, with 1771 SSRs (89.99%) being mono-nucleotide type, particularly A and T repeat motifs. In the di-nucleotide repeat type, 176 SSRs were AT/TA repeat motifs. Only one TC and AG repeat motifs were found in *H. ovalifolia*, *Dorcoceras hygrometricum*, *Litostigma coriaceifolium* Y. G. Wei, F. Wen & Mich. Möller, *Primulina lobulata* (W.T. Wang) Mich. Möller & A. Weber and *Primulina xiziae* F. Wen, Yue Wang & G. J. Hua, respectively. In tri-nucleotide repeat type, only TTA, ATA and ATT repeat motifs were found in *Paraboea filipes* (Hance) Burtt*, P. peltifolia* D. Fang & L. Zeng, *P. clavisepala* D. Fang & D. H. Qin, *P. dictyoneura* (Hance) Burtt, *P. dolomitica* Z.Y. Li, X.G. Xiang & Z.Y. Guo and *Primulina fimbrisepala* (Hand.-Mazz.) Yin Z. Wang. Similarly, in the tetra-nucleotide repeat type, only one ATCT repeat motif was found in *H. malipoensis* Y. H. Tan, *H. sinovietnamica, H. subacaulis* Hand.-Mazz. *Petrocodon jingxiensis* (Yan Liu, H. S. Gao & W. B. Xu) A. Weber & Mich. Möller, and *Primulina linearifolia* (W. T. Wang) Yin Z. Wang. A total of 2055 tandem repeats were detected, ranging from 13 (*Petrocodon longitubus* Cong R. Li & Yang Luo) to 47 (*Corallodiscus lanuginosus*). There were 2802 dispersed repeats in the Trichosporeae plastomes, of which 1185 (42.29%) were forward repeats, 1502 (53.60%) were palindromic repeats, 86 (3.07%) were reverse repeats, and 29 (1.03%) were complement repeats.

### 3.3 Codon usage bias

The codon usage bias of CDSs in Trichosporeae was shown in **Figures S2, 3**. There were 30 preferred codons (RSCU>1), 2 non-preferred codons (RSCU=1), and 32 less frequently used codons (RSCU<1). The preferred codons typically ended with A or U, except for UUG. Moreover, leucine (Leu, encoded by UUA, UUG, CUU, CUC, CUA and CUG) was the most frequently encoded amino acid, while cysteine (Cys, encoded by UGU and UGC) was the least frequently encoded amino acid. The two non-preferred codons (Met and Trp) only had a unique codon. Within Trichosporeae, the ENC values of CDSs ranged from 25.17 to 61. Remarkably, the ENC values of *psbI* in 78 species (except *Corallodiscus lanuginosus*), *petN* in 74 species (except *Corallodiscus lanuginosus, Litostigma coriaceifolium*, *Paraboea filipes* (Hance) Burtt, *Petrocodon longitubus* and *Primulina renifolia* (D.Fang & D.H.Qin) J.M.Li & Yin Z.Wang), and *psbJ* in 77 species (except *Corallodiscus lanuginosus* and *Primulina huaijiensis* Z. L. Ning & J. Wang) were less than 35, indicating significant codon bias for these genes.

### 3.4 Phylogenetic reconstruction

**Figure 5:**
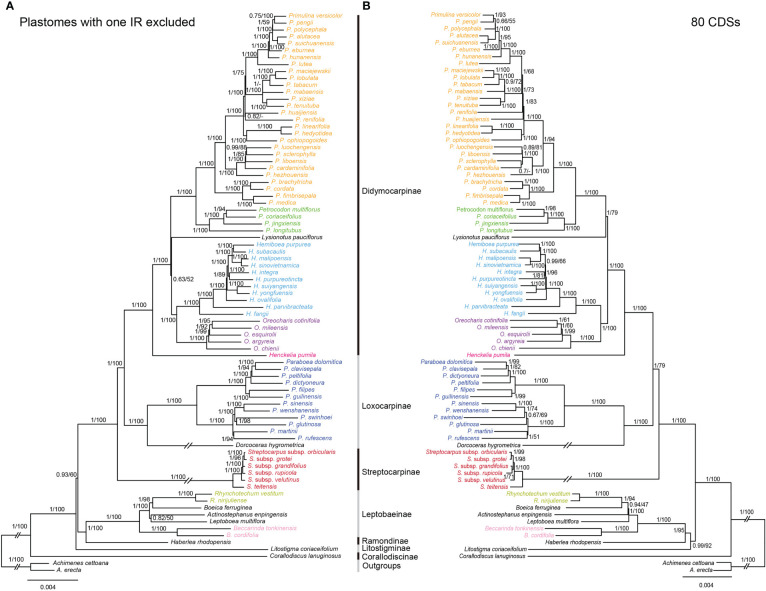
Comparisons of phylogenetic topologies for plastomes with one IR excluded **(A)** and 80 CDSs **(B)** based on BI and ML analyses in Trichosporeae species. Numbers above the branches are Bayesian posterior probabilities and ML bootstrap values, respectively. A dash (-) indicates that the supporting values are less than 50%.

The topologies based on the whole plastome (excluding one IR) and 80 CDs were basically concordant, with both showing strong support for the monophylies of the 7 sampled subtribes and 17 genera (**Figure 5**). BI and ML analyses yielded nearly identical topologies, with some differences in the supporting values of certain nodes. The whole plastome had a higher overall resolution power compared to the 80 CDSs. The subtribe Corallodiscinae diverged first, followed by Litostigminae. Ramondinae and Leptobaeinae formed a sister group (PP_BI_=1.00, BS_ML_=100%). Loxocarpinae and Didymocarpinae were strongly supported as a sister group (PP_BI_=1.00, BS_ML_=100%) (**Figure 5A**). Within subtribe Didymocarpinae, *Henckelia pumila* (D. Don) A. Dietr. diverged first (PP_BI_=1.00, BS_ML_=100%). *Oreocharis* formed a sister group of *Hemiboea* with high supporting values (PP_BI_=1.00, BS_ML_=100%). However, the phylogenetic position of *Lysionotus* could not be determined.

### 3.5 Evolution of morphological characters

The ancestral reconstructions of the nine morphological characters were shown in **Figure S4**. The results indicated that the evolution patterns of morphological characters in Trichosporeae were complex. Some characters, such as habit, inflorescence and seeds, remain constant or nearly similar within tribe. However, some generic diagnostic morphological characters, including stem, leaf margin, numbers of stamens and staminodes, capsule, and its dehiscing type, have evolved several times independently within tribe. Additionally, the leaf margin has shifted several times among *Primulina* species.

## 4 Discussion

### 4.1 Plastome evolution within Trichosporeae

Plastomes in angiosperms are generally maternal inherited and exhibit a highly conserved structure among different plant lineages (Wicke et al., 2011). In this study, the whole plastome sequences of 11 *Hemiboea* species were obtained and found to be very similar in size (152,742–153, 695 bp), structure, gene order, gene content, and GC content (**Table S2; Figures S1**). The high conservatism of plastome has also been observed in other Gesneriaceae genera. For instance, Wang et al. (2022) found that the plastomes length of *Paraboea* ranged from 153,166–154,245 bp and exhibited similar structure, gene order, and gene content. Additionally, our results showed that the GC content of the IR region (43.1–43.2%) was higher than that of the LSC region (35.5–35.6%) and the SSC region (30.9–31.2%).

Within Trichosporeae, no contraction or expansion of IR borders was detected. However, there was relatively larger plastome variation in gene content (121–133), plastome length (152,196–156,614 bp), and GC content (37.2–37.8%) (**Tables S2**). Higher GC content is usually related to the stability of the DNA strands, which might be due to the presence of rRNA and tRNA in the IR region (Necşulea and Lobry, 2007; Ding et al., 2021). In this study, 30 preferred codons were found within Trichosporeae, all ending with A or U except for UUG (**Figure S2**). The codon usage patterns of plastomes were relatively conserved and highly similar over long evolutionary periods, possibly due to natural selection and mutation (Necşulea and Lobry, 2007; Sharp et al., 2010). Among the 20 amino acids, *Cys* was the least frequently encoded amino acid (**Figure S2**), possibly due to the gene being highly sensitive to physiological and environmental conditions (Marino and Gladyshev, 2012). Codon bias is crucial for understanding species evolution and predicting gene function and expression levels (Uddin et al., 2015).

SSRs are very often used as genetic molecular markers in phylogenetic studies of closely related species (e.g., Cheng et al., 2005; Angioi et al., 2009; Ebert and Peakall, 2009). In this study, a total of 1968 SSRs were identified in the plastomes of 79 Trichosporeae species, with 89.99% of them being mono-nucleotide repeats (**Figure 4**; **Table S4**). These results are consistent with previous studies of other angiosperms (Xiang et al., 2022; Yu et al., 2022). Plastome SSRs usually consist of short polyA or polyT repeats and mono-nucleotide repeats (Provan et al., 2001). The variation in the non-coding region was higher than that in the coding region, with the variation in the SC region being higher than that in the IR region, which is like other angiosperms plastomes (Xiang et al., 2022). Five hypervariable CDSs (*rps15*, *matK*, *ndhF*, *rps16* and *rpl32*) and eight hypervariable non-coding regions (*rpl16/rps3*, *rpl14/rpl16*, *petB/petD*, *trnC-GCA/petN*, *rps15/ycf1*, *trnfM-CAU/rps14*, *petD/rpoA* and *trnS-GGA/rps4*) were identified in Trichosporeae (**Figure 3**). Among these, *ndhF*, *matK* and *rps16* have been employed in previous phylogenetic studies (Roalson and Roberts, 2016; Li et al., 2022). Other hypervariable regions, such as *rps15*, *rpl32, rpl16/rps3*, *rpl14/rpl16*, *petB/petD*, *trnC-GCA/petN*, *rps15/ycf1*, *trnfM-CAU/rps14*, *petD/rpoA* and *trnS-GGA/rps4*, have great potential to be exploited as DNA barcodes markers, which might be used for further species identification. SNPs and indels play an important role in elucidating genome evolution (Matsuoka et al., 2002; Grover et al., 2008). In Trichosporeae, the majority of SNPs from coding genes were functionally missense variations, indicating a relatively complex evolutionary history.

### 4.2 Phylogenomics and morphological evolution within Trichosporeae

Previously, the phylogenetic relationships within Trichosporeae were unclear based on *trnL-F* and ITS (Möller et al., 2011). Although Roalson and Roberts (2016) and Li et al. (2022) obtained more molecular data, the relationships among subtribes and genera have not been resolved. In this study, the most extensive phylogenetic relationships of Trichosporeae were established (**Figure 5**). Our study strongly supported the monophyly of seven sampled subtribes and 17 genera (**Figure 5**). Also most relationships among subtribal level and genera were clarified with strong support based on the whole plastomes without one IR (**Figure 5**). The sister relationships between Loxocarpinae and Didymocarpinae were confirmed here (PP_BI_=1.00, BS_ML_=100%), which was consistent with Li et al. (2022). Furthermore, *Oreocharis* was found to be a sister group of *Hemiboea* with high supporting values (PP_BI_=1.00, BS_ML_=100%). However, the phylogenetic positions of *Lysionotus* remain controversial based on whole plastomes (PP_BI_=0.63, BS_ML_=52%) and 80 CDSs (PP_BI_=1.00, BS_ML_=79%). This controversial result was also found in previous studies. For example, the phylogenetic relationships among *Lysionotus*, *Hemiboea*, *Oreocharis*, *Primulina* and *Petrocodon* were unclear based on whole plastomes (Xu et al., 2022). Gu et al. (2020) showed that *Lysionotus* was a sister group of *Oreocharis* based on whole plastomes (PP_BI_=0.68, BS_ML_=44%). However, Wen et al. (2022) showed that *Lysionotus* was close related to *Primulina* and *Petrocodon* based on 80 CDSs (PP_BI_=0.87, BS_ML_=88%). These findings might contribute to future research on systematic analysis, genetic diversity and evolutionary patterns of the tribe Trichosporeae, and support the use of plastome to resolve the phylogenetic relationships of different taxonomic levels.

The Trichosporeae is characterized by morphological heterogeneity and is considered the most taxonomically difficult group (Weber et al., 2013). While traditional classifications based on morphological characters recognized genera in different alliances and geographical groups, recent phylogenomic data have almost clarified the relationships among subtribes. Our results showed that, except for conserved vegetative characters, Trichosporeae is greatly divergent with regard to its floral and capsule characters (**Figure S4**). For instance, the ancestors of this tribe had 2 stamens, which independently evolved into 4 stamens in *Oreocharis* and Leptobaeniae; the numbers of staminodes have evolved several times and even diverged in genus *Primulina*. Weber et al. (2013) considered that the advanced Asiatic and Malesian genera of this tribe were divided into a comparatively small group of genera with predominantly twisted fruits and a much larger group with straight fruits. However, our results inferred that the ancestor groups had a straight capsule, and the twisted capsule had independently evolved at least three times in different subtribes. Therefore, the complex morphological character evolution of Trichosporeae would be significant in defining larger groups and understanding their wide distribution.

## 5 Conclusion

In this study, we reported eleven new *Hemiboea* plastome sequences. Comparative analysis revealed that Trichosporeae plastomes were relatively conserved in genome structure, gene order, gene content, GC content and codon usage bias. Contraction and expansion of IR borders were not detected, and the 13 hypervariable regions were identified as potential molecular markers for species identification in Trichosporeae. We identified 1968 simple sequence repeats, 2055 tandem repeats and 2802 dispersed repeats. Our results highly confirmed the monophyly of seven sampled subtribes and 17 genera. We clarified the sister relationships between Loxocarpinae and Didymocarpinae, and supported the close relationship between *Oreocharis* and *Hemiboea* with highly supported values. Furthermore, the morphological character evolution was complex, and Trichosporeae was greatly divergent in its floral and capsule characters. Future research should employ more samples and molecular markers to investigate the phylogenetic relationships and comprehensively infer the complex evolutionary history of Trichosporeae based on extensive morphological characters.

## Data availability statement

The datasets presented in this study can be found in online repositories. The names of the repository/repositories and accession number(s) can be found below: https://www.ncbi.nlm.nih.gov/genbank/, OP820508-OP820512, OQ799915-OQ799920.

## Author contributions

X-GX and Z-YL conceived and designed the study. X-GX, QZ, BP, Y-SH, and Z-YG did fieldwork. Y-FC, PZ, HY, and L-GZ conducted experiments and data analysis. Y-FC, X-GX, K-LX, and PZ wrote the draft. All authors revised the manuscript. All authors contributed to the article and approved the submitted version.
