# Supplementary material for: Plastome evolution and phylogenomics of Trichosporeae (Gesneriaceae) with its morphological characters appraisal
Source: Front Plant Sci. 2023 May 9;14:1160535. doi: 10.3389/fpls.2023.1160535 (PMC10203511; doi:10.3389/fpls.2023.1160535)
Supplement: Supplementary file 1 [file DataSheet_1.zip › SI/Cui et al., SI clear.docx]

**Supplementary materials**

**Table S1** Taxa, source and GenBank accession numbers used in this study.

**Table S2** Summary of plastome characters among 79 Trichosporeae species.

**Table S3** Group of genes within the Trichosporeae plastomes.

**Table S4**  Numbers of trandem repeat, simple sequence repeat and dispersed repeat in 79 Trichosporeae plastomes.

**Figure S1** Visualization of the collinearity. Structural alignment of 79 Trichosporeae (Didymocarpoideae) plastomes using progressive Mauve following the topology of the ML tree based plastomes with one IR excluded. The inverted repeat region B (IRB) was removed from the analysis. The lines linking the collinear blocks represent homology between different genomes. Numbers on the upper x-axis are genome map coordinates in kilobases (Kb).

**Figure S2** Heat map of codon usage bias in the plastomes of Trichosporeae under RSCU.

**Figure S3** Heat map of codon usage bias in the plastomes of Trichosporeae under CDSs.

**Figure S4** Ancestral state reconstruction of nine morphological characters. A. habit, B. stem, C. leaf margin, D. inflorescence, E. number of stamens, F. number of staminodes, G. capsule, H. capsule dehiscing type, and I. seeds.
