# Supplementary figures and images for: Plastome evolution and phylogenomics of Trichosporeae (Gesneriaceae) with its morphological characters appraisal
Source: Front Plant Sci. 2023 May 9;14:1160535. doi: 10.3389/fpls.2023.1160535 (PMC10203511; doi:10.3389/fpls.2023.1160535)

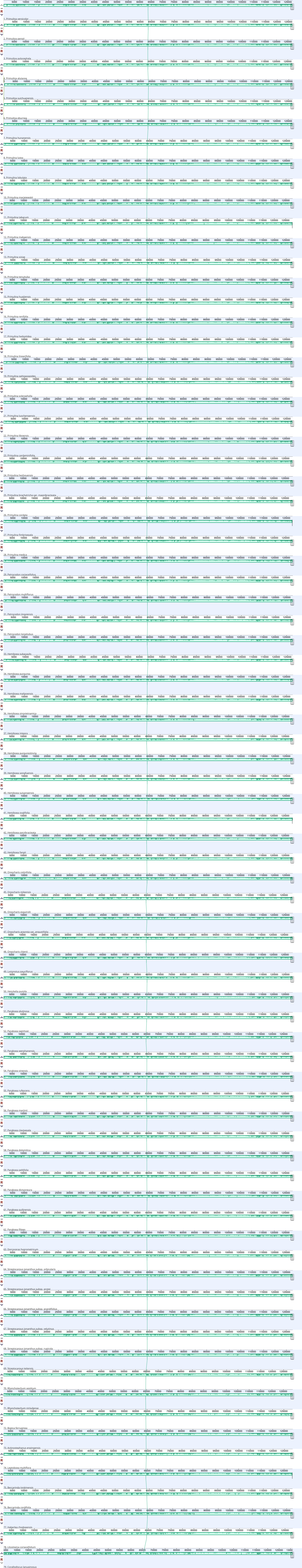

Supplement: Supplementary file 1 [file DataSheet_1.zip › SI/Cui et al., Figures S1.jpg]

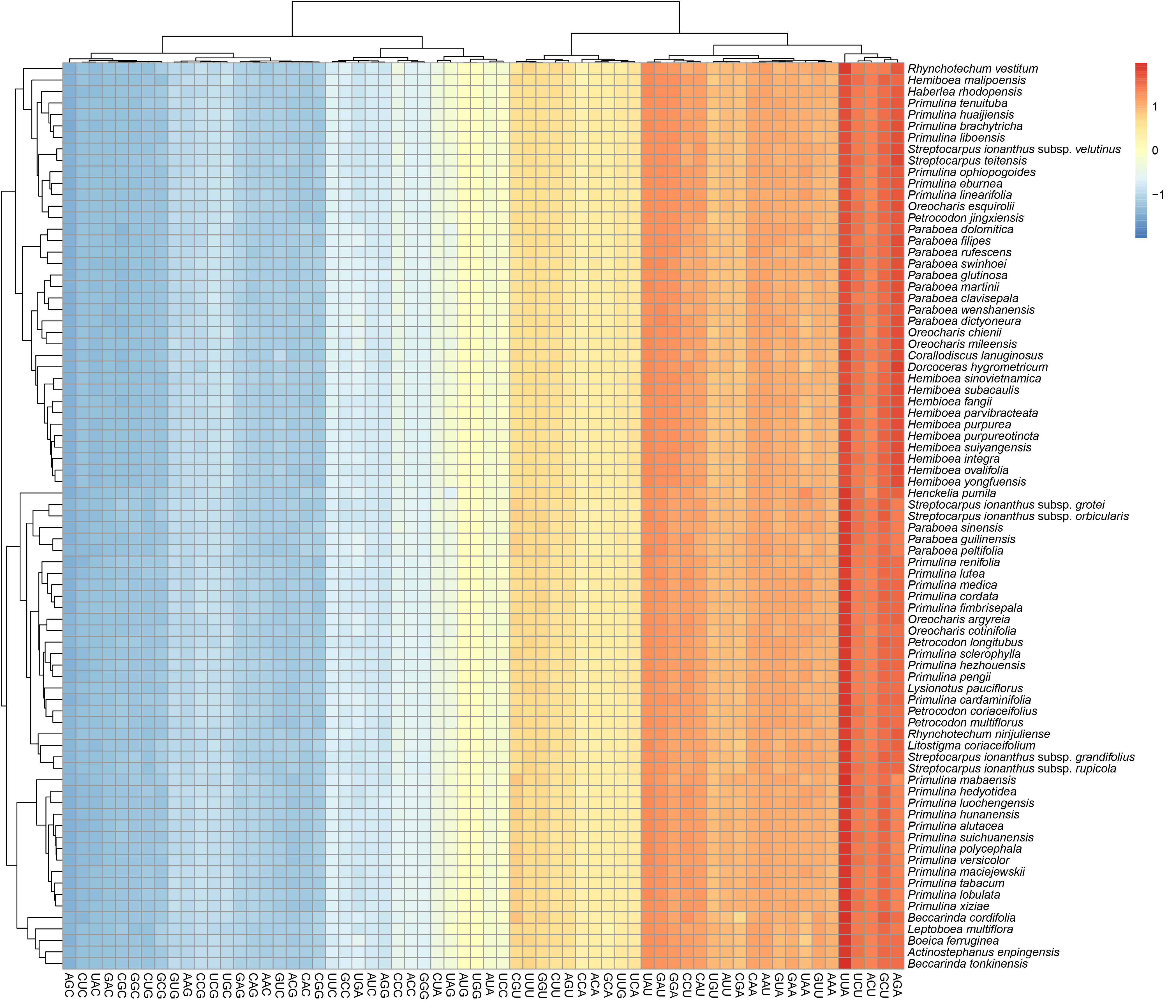

Supplement: Supplementary file 1 [file DataSheet_1.zip › SI/Cui et al., Figures S2.jpg]

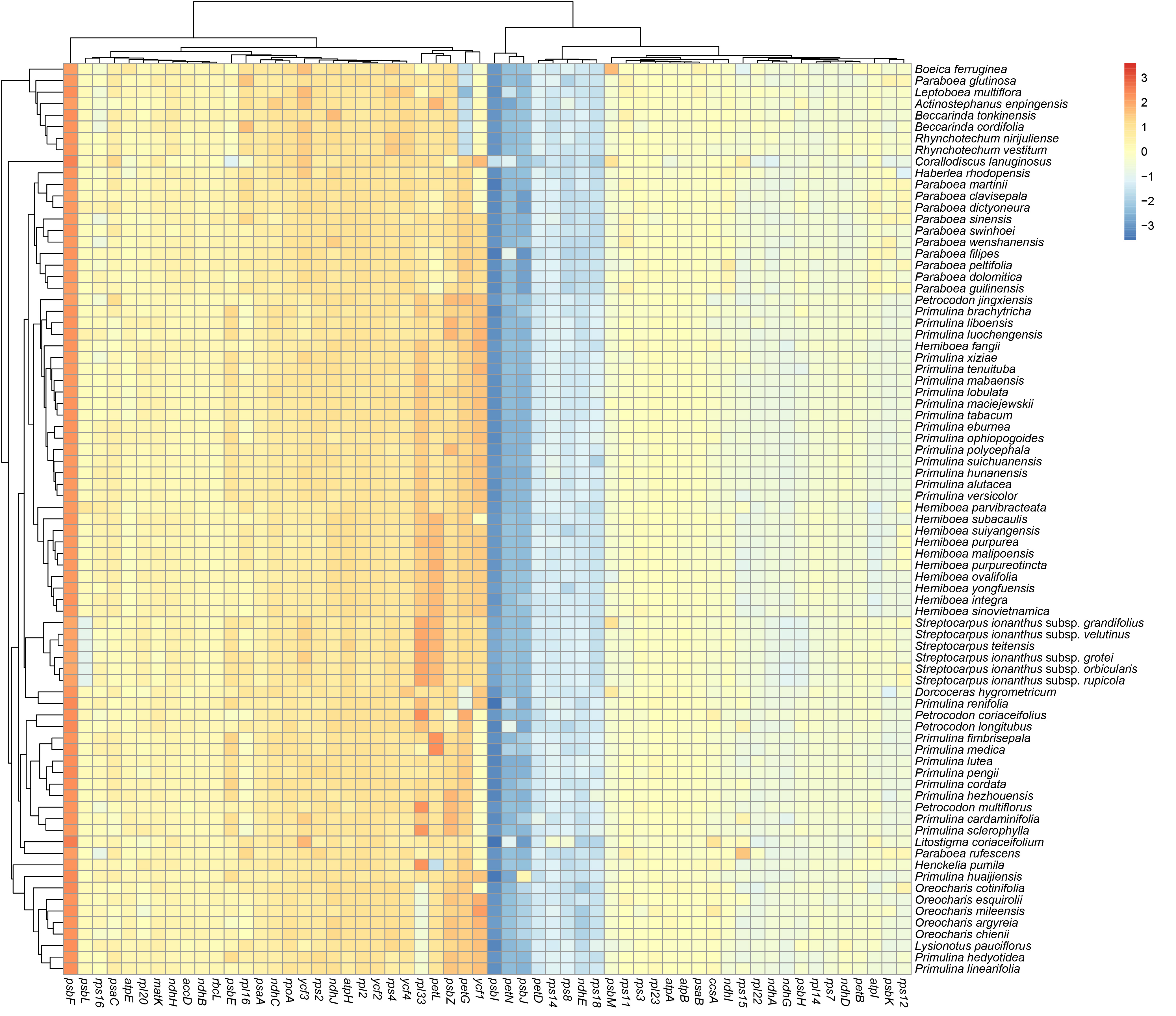

Supplement: Supplementary file 1 [file DataSheet_1.zip › SI/Cui et al., Figures S3.jpg]

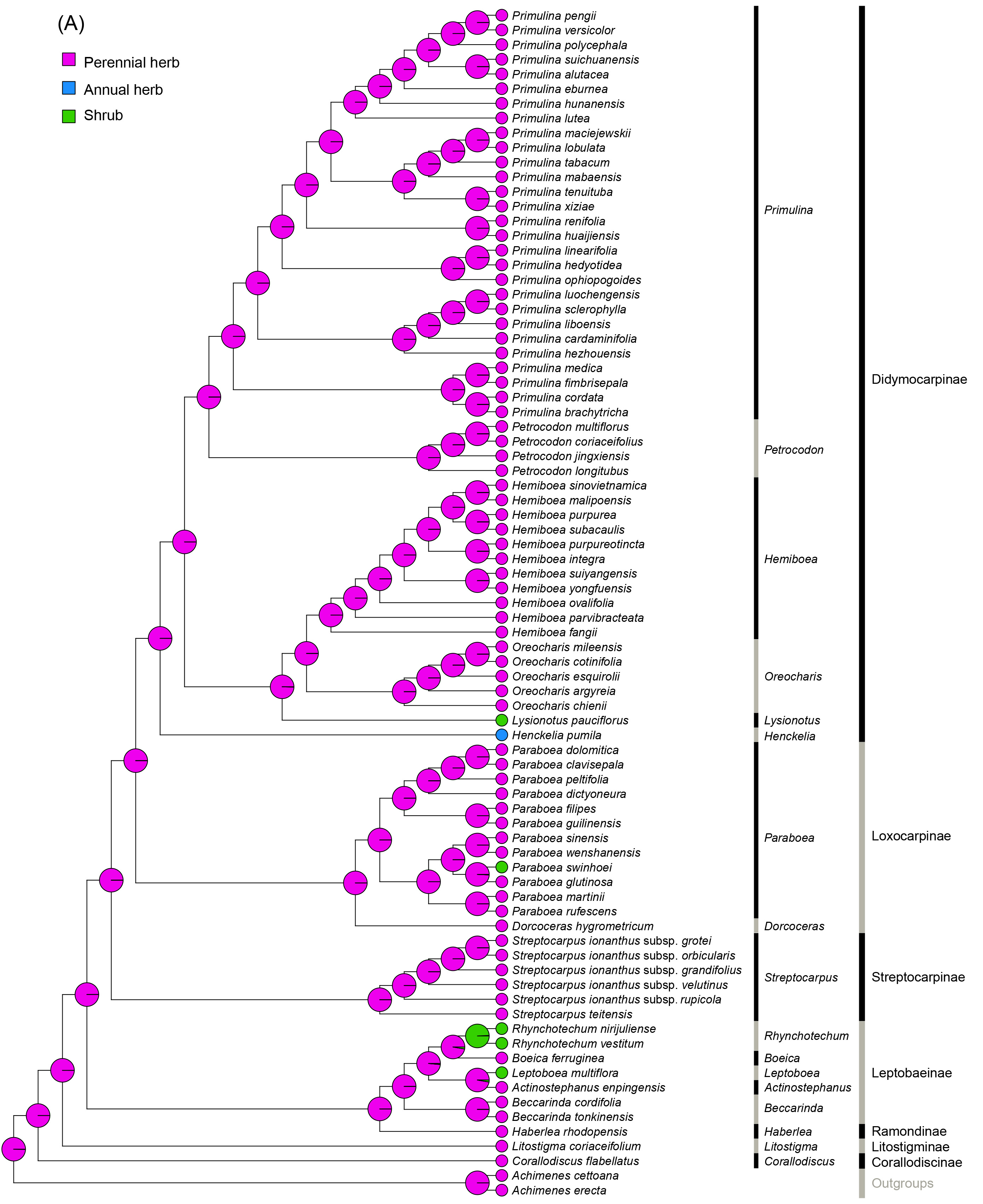

Supplement: Supplementary file 1 [file DataSheet_1.zip › SI/Cui et al., Figures S4A.jpg]

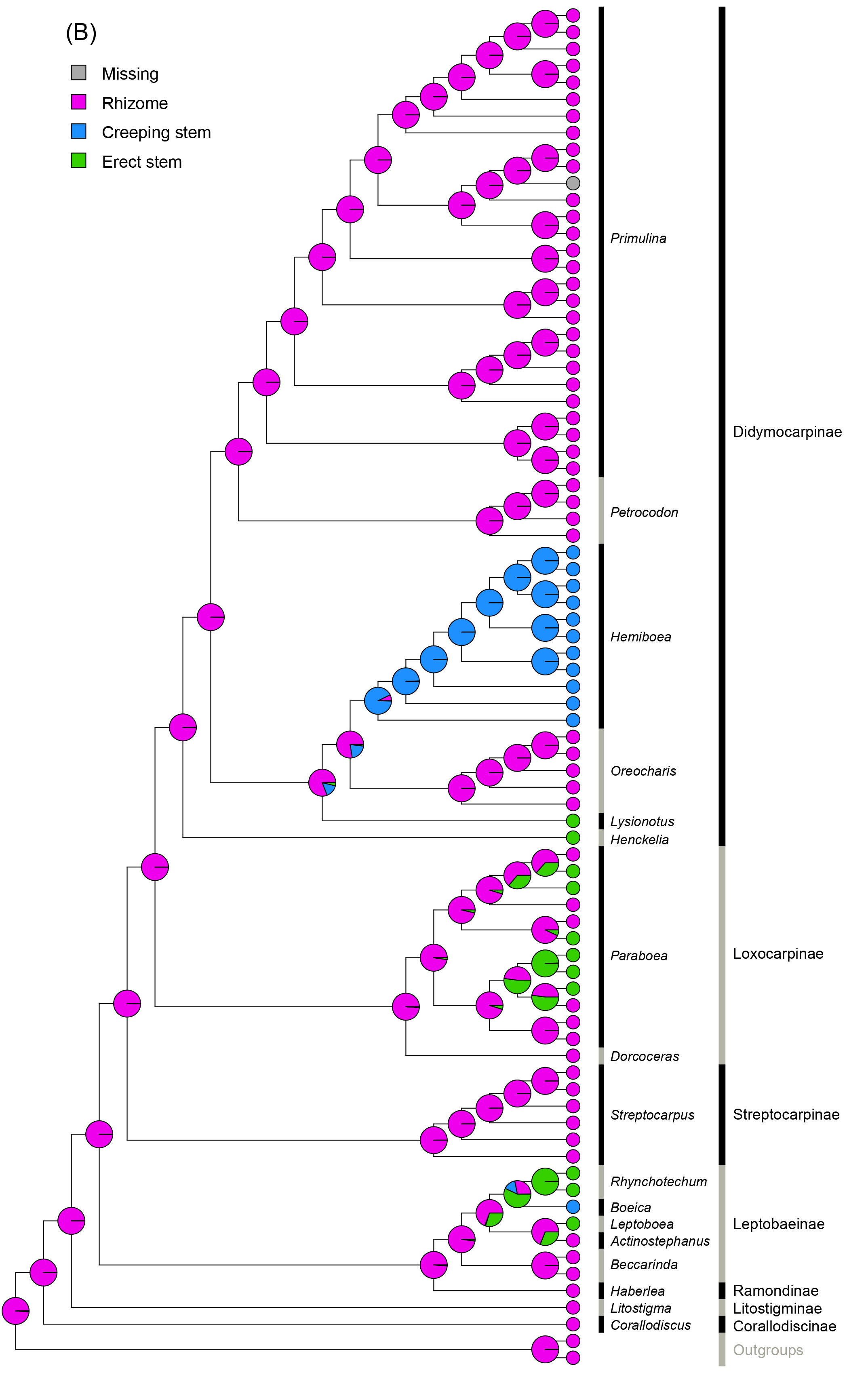

Supplement: Supplementary file 1 [file DataSheet_1.zip › SI/Cui et al., Figures S4B.jpg]

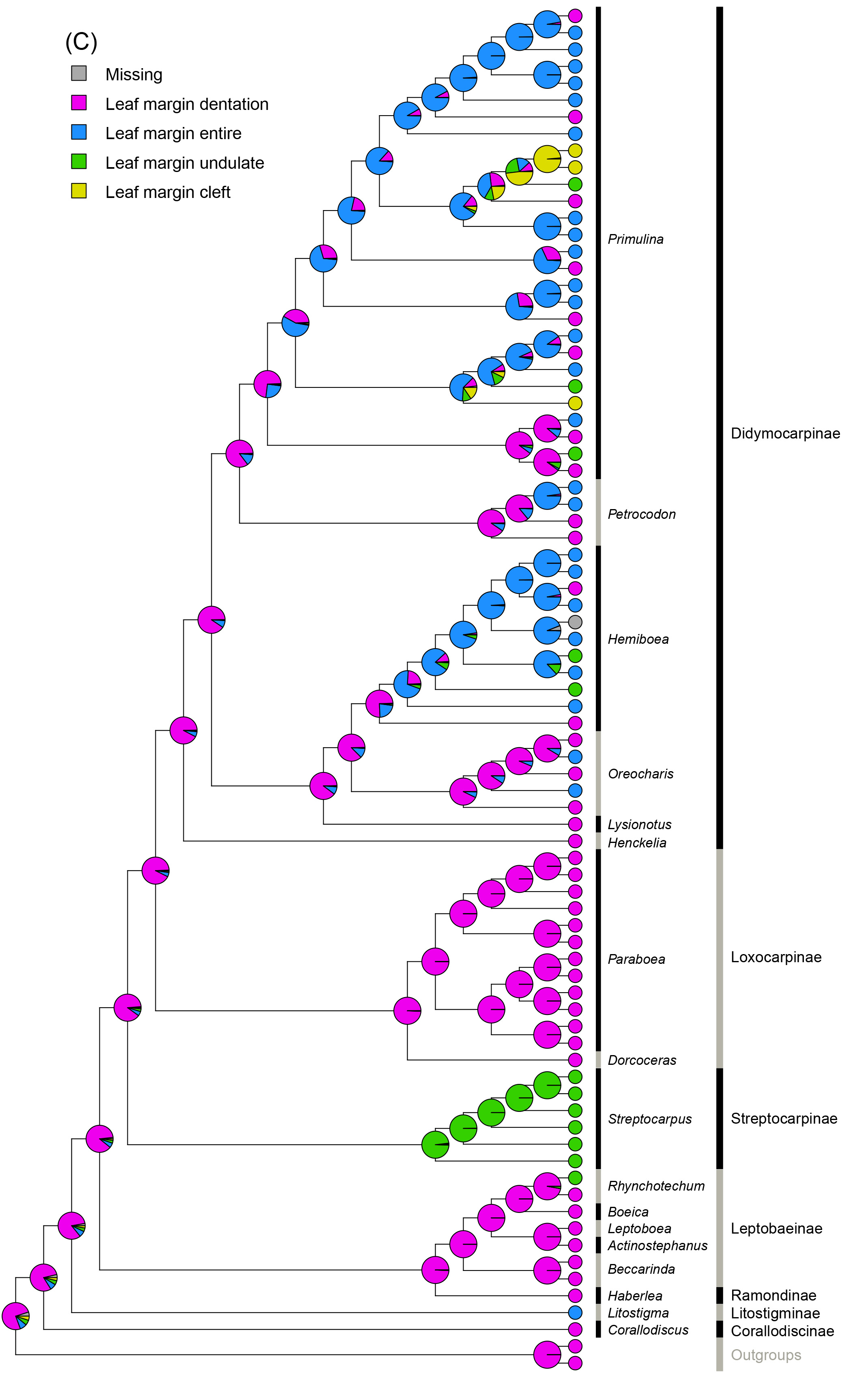

Supplement: Supplementary file 1 [file DataSheet_1.zip › SI/Cui et al., Figures S4C.jpg]

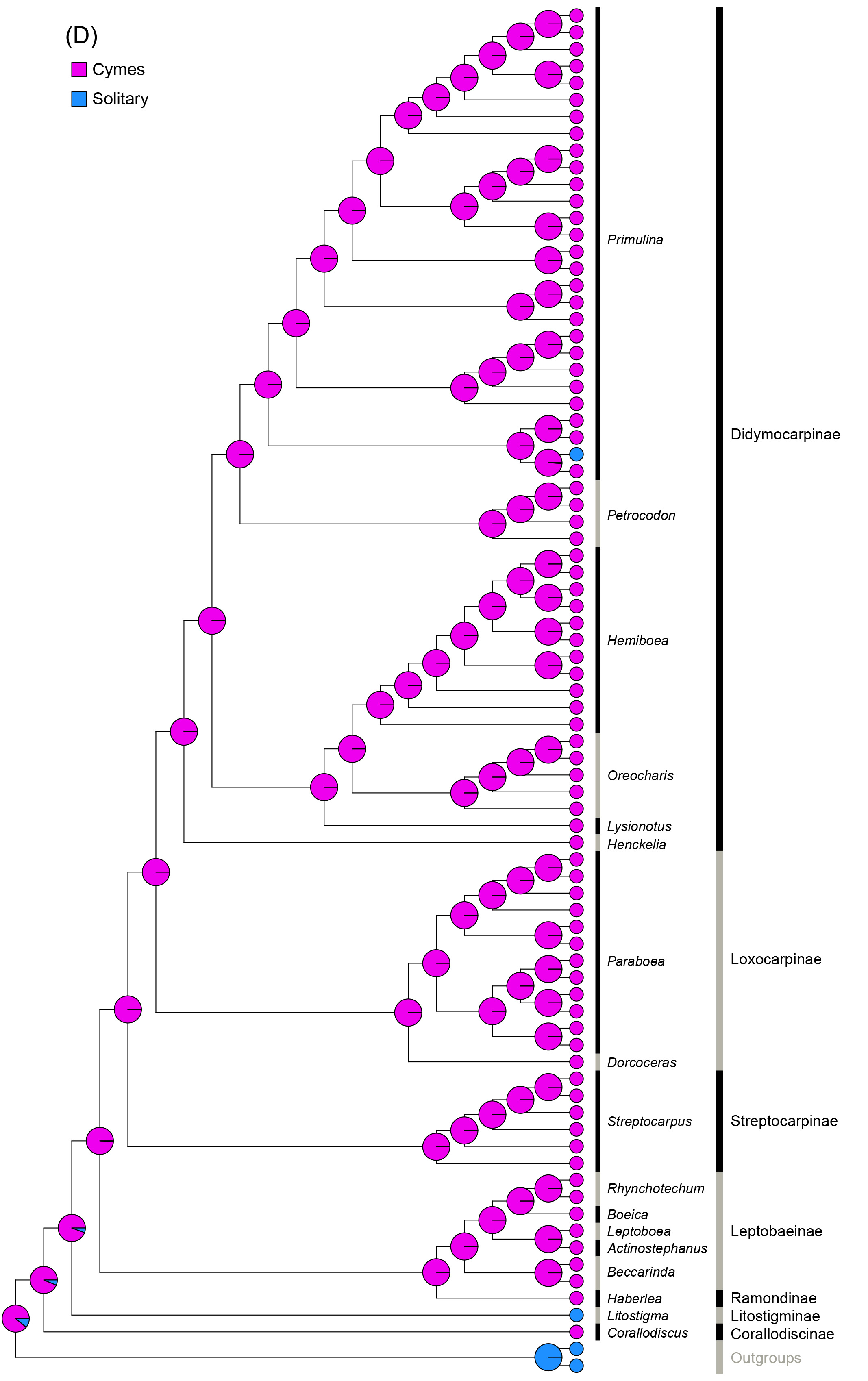

Supplement: Supplementary file 1 [file DataSheet_1.zip › SI/Cui et al., Figures S4D.jpg]

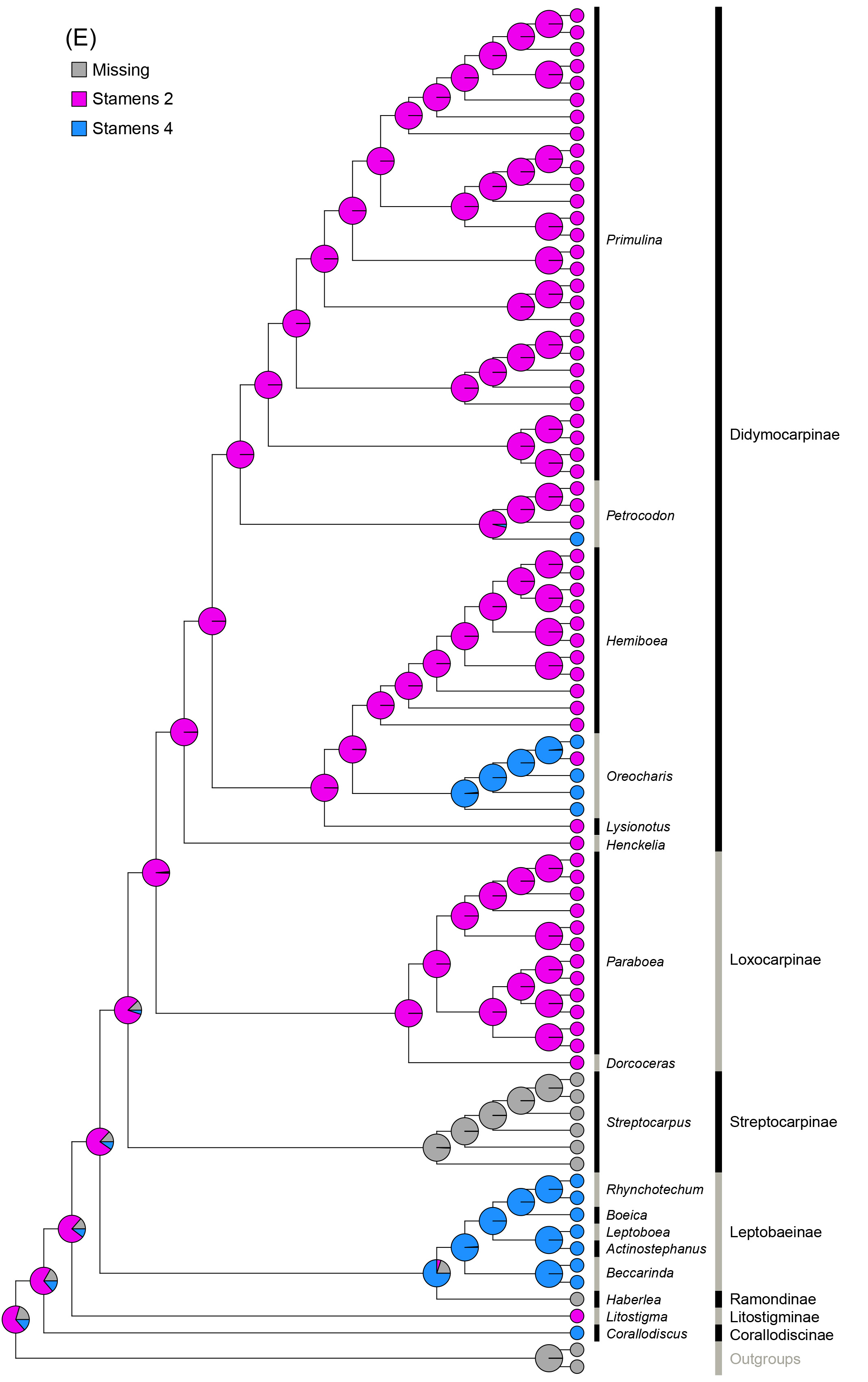

Supplement: Supplementary file 1 [file DataSheet_1.zip › SI/Cui et al., Figures S4E.jpg]

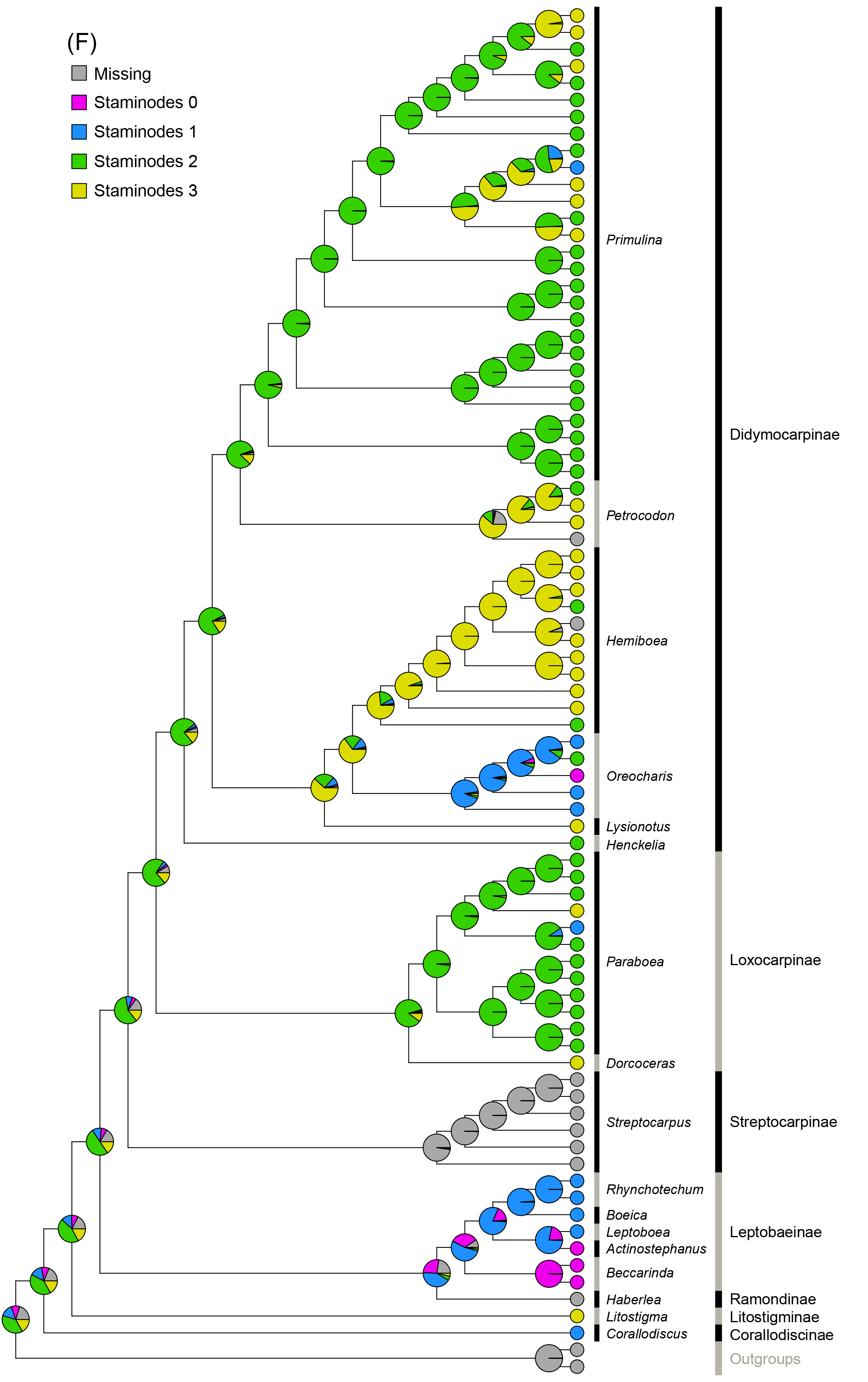

Supplement: Supplementary file 1 [file DataSheet_1.zip › SI/Cui et al., Figures S4F.jpg]

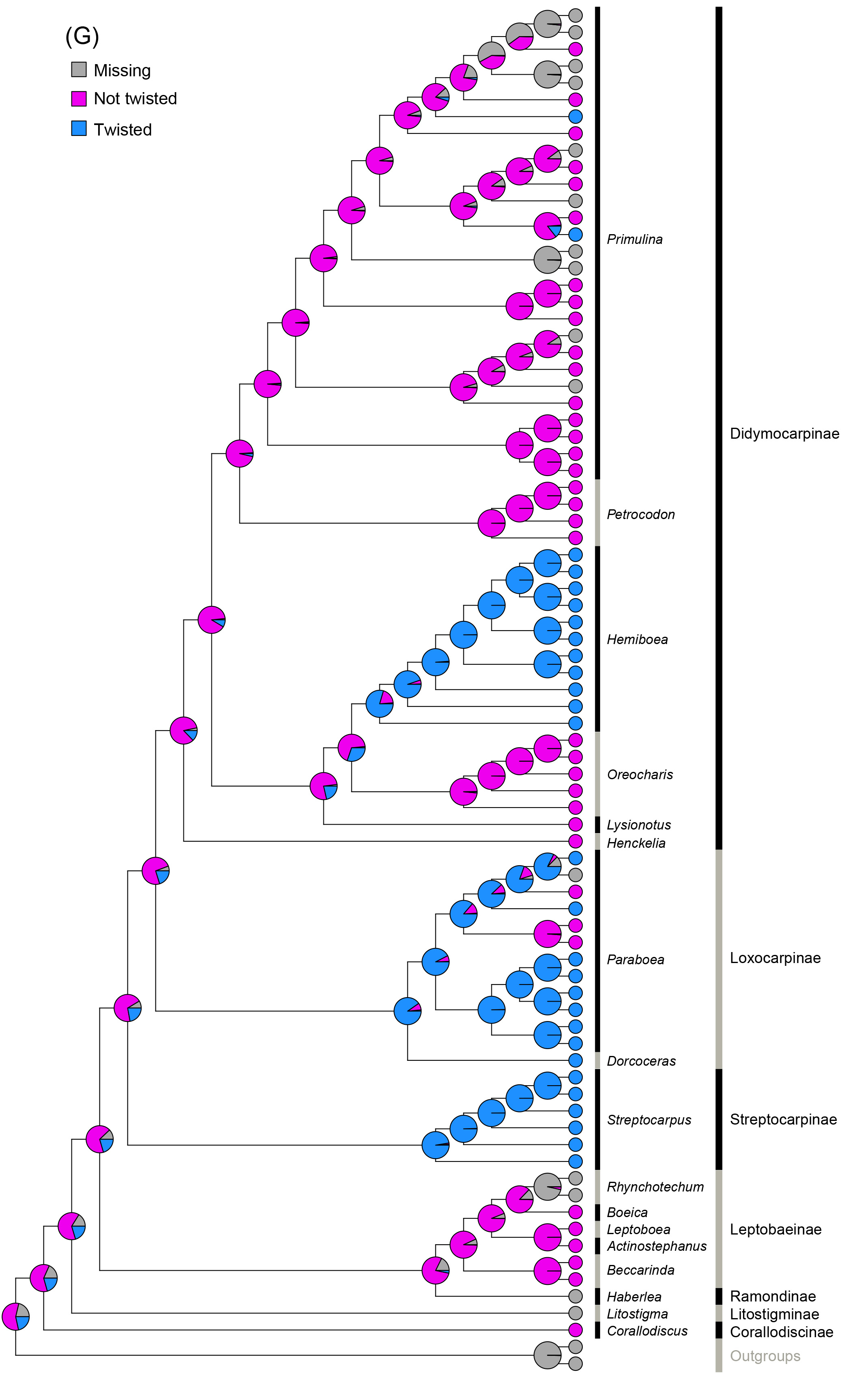

Supplement: Supplementary file 1 [file DataSheet_1.zip › SI/Cui et al., Figures S4G.jpg]

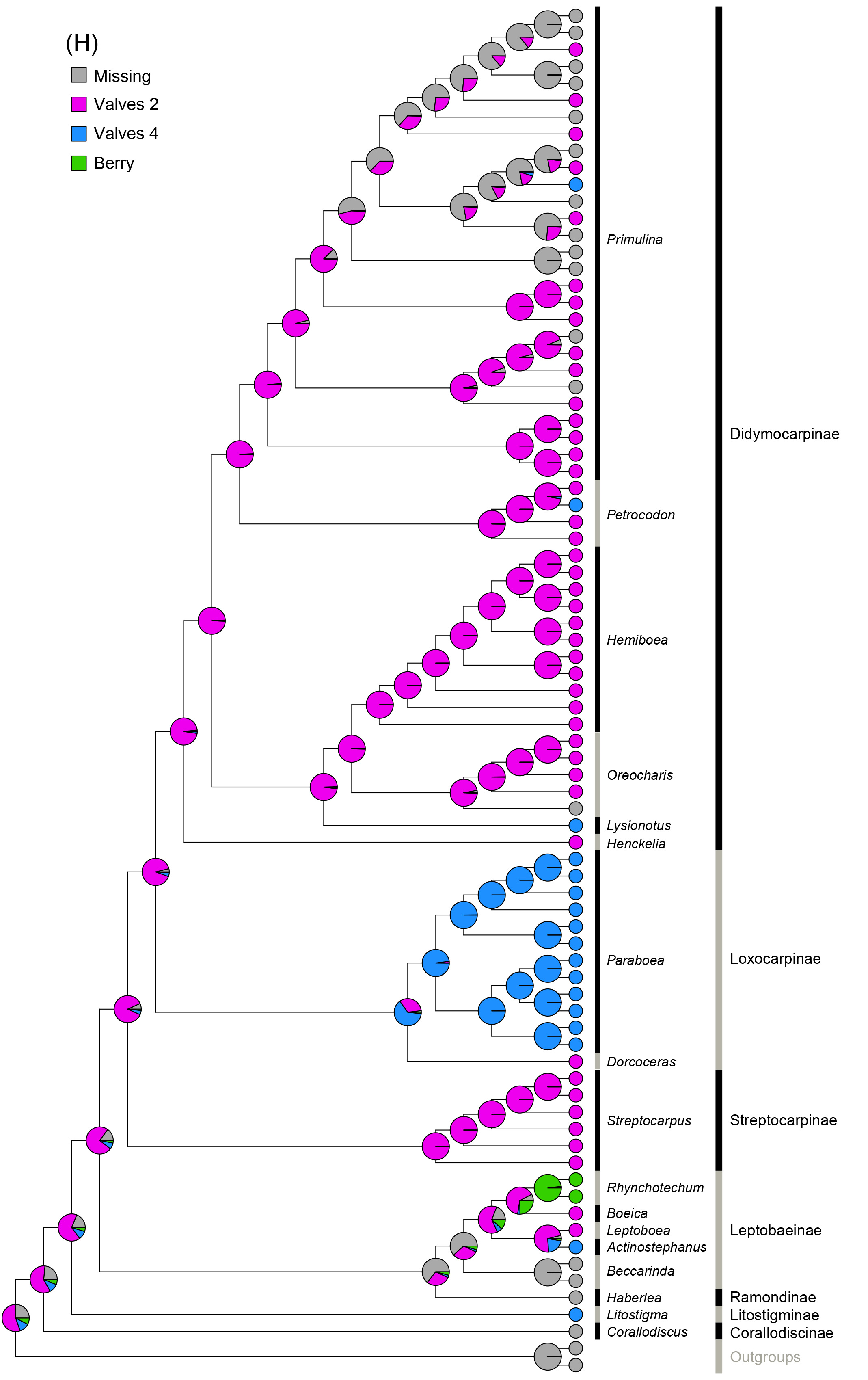

Supplement: Supplementary file 1 [file DataSheet_1.zip › SI/Cui et al., Figures S4H.jpg]

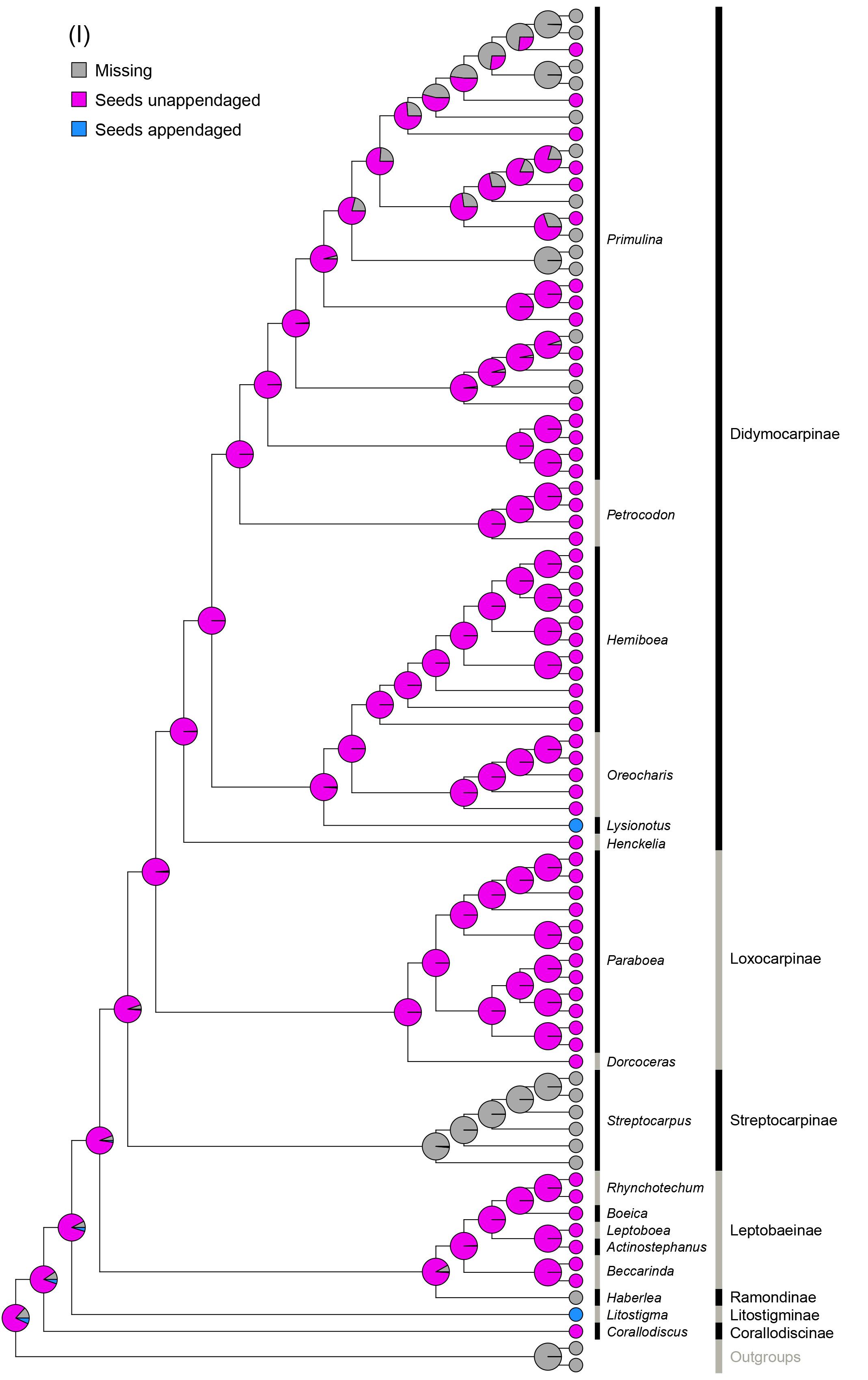

Supplement: Supplementary file 1 [file DataSheet_1.zip › SI/Cui et al., Figures S4I.jpg]
